# Obesity Among High School Students in the United States: Risk Factors and Their Population Attributable Fraction

**DOI:** 10.5888/pcd15.180122

**Published:** 2018-11-08

**Authors:** Edward Y. Hu, Sujith Ramachandran, Kaustuv Bhattacharya, Sasikiran Nunna

**Affiliations:** 1 Oxford High School, Oxford, Mississippi; 2 Department of Pharmacy Administration, the University of Mississippi School of Pharmacy, University, Mississippi

## Abstract

**Introduction:**

The prevalence of obesity among children and adolescents in the United States is high. The aim of this study was to assess the association between modifiable risk factors and obesity and to estimate the population attributable fractions (PAFs) of modifiable risk factors among high school students in the United States.

**Methods:**

For this retrospective study, we used a nationally representative sample of 15,624 students who participated in the 2015 Youth Risk Behavior Survey (YRBS). Obesity was defined as body mass index at or above the 95th percentile, based on sex- and age-specific data from the Centers for Disease Control and Prevention. We examined unhealthy dietary behaviors, physical inactivity, and other modifiable risk factors (tobacco use, alcohol consumption, and sleep). We used multivariable logistic regression, accounting for the complex survey design of YRBS, to assess the association between risk factors and obesity and to calculate PAFs. Confidence intervals of PAFs were estimated by using the jackknife repeated replication method.

**Results:**

Among all students included in the study, 13.9% were classified as obese. Not being on a sports team (odds ratio [OR], 1.61; 95% confidence interval [CI], 1.31–1.98), current tobacco use (OR, 1.42; 95% CI, 1.14–1.77), and watching television for 3 hours or more per day (OR, 1.38; 95% CI, 1.09–1.76) were significantly correlated with obesity. The combined PAF for all modifiable risk factors was 34.80% (95% CI, 32.09%–37.51%). The single modifiable risk factor with the largest PAF was not participating on a sports team (PAF, 16.57%; 95% CI, 15.30%–17.84%).

**Conclusion:**

Findings about PAFs help demonstrate the importance of promoting physical activity, healthy diet, and other healthy lifestyles in reducing obesity among high school students in the United States.

## Introduction

The prevalence of obesity among children and adolescents in the United States increased from 10.6% to 13.9% during 1999 through 2015 (1). Obesity can lead to serious adverse consequences such as asthma, obstructive sleep apnea, joint problems, hypertension, hypercholesterolemia, low self-esteem, and depression (2–7). Furthermore, children with obesity are 5 times more likely to be obese in adulthood, leading to long-term morbidity and mortality (8). The common risk factors of obesity may or may not be modifiable. Factors such as genetic variation, ethnic origin, and birth weight are not modifiable, whereas other factors such as dietary intake, physical activity, and sedentary behaviors (eg, watching television or using other screen devices) are modifiable (5–7,9–11).

Although it is challenging to determine the exact cause of obesity in any individual, efforts aiming to quantify the contribution of modifiable risk factors to childhood and adolescent obesity would help to prioritize prevention and treatment strategies to reduce such obesity. This evidence can be generated by using population attributable fractions (PAFs), defined as the proportion of disease or condition (eg, obesity) that could be prevented if a risk factor (eg, sedentary lifestyle) were removed from the population (12–15). Childhood and adolescent obesity prevalence is usually estimated through national surveys that employ complex survey designs (1,4,16), but statistical packages such as SAS (SAS Institute Inc) or Stata (StataCorp LLC) do not support the estimation of PAF in complex survey designs (13,17). To overcome this challenge, we used a statistical tool with a SAS macro developed by Heeringa et al (17) to estimate PAFs from complex sample survey data to assess the association between modifiable risk factors and determine the PAFs of modifiable risk factors of obesity in a nationally representative sample of high school students in the United States.

## Methods

### Study design and data

This study was a retrospective cross-sectional analysis of national data from the 2015 Youth Risk Behavior Survey (YRBS) of high school students (18). The national high school YRBS is conducted by the Centers for Disease Control and Prevention (CDC) to estimate the prevalence of health risk behaviors among US high school students. To achieve a nationally representative sample of students in public and private schools in grades 9 through 12, YRBS employs a complex sample scheme and a 3-stage cluster sample design with oversampling of certain subgroups such as black and Hispanic students. To protect students’ privacy, survey participation is anonymous and voluntary (1).

The 2015 YRBS public data file contains 15,624 usable questionnaires; the survey had an overall response rate of 60%. Weighting procedures were applied to each record in the national YRBS to adjust for nonresponse and oversampling, making the weighted estimates representative of all US students in grades 9 through 12 attending public and private schools (1). The 2015 YRBS data used for this study were de-identified and publicly available; therefore, a review by the institutional review board was waived.

### Measures

The individual student’s obesity status was the dependent variable in this study. Obesity was defined as having a body mass index calculated from self-reported height and weight (ie, weight in kilograms divided by height in meters squared) at or above the 95th percentile based on sex- and age-specific reference data from the 2000 CDC growth charts (19). We treated respondent’s obesity status as a dichotomous variable (obese or not obese).

The selection of modifiable risk factors for this study was informed by literature and the availability of information in YRBS data (1,6,9,20,21). We categorized the identified risk factors as being related to diet, physical activity, or other lifestyle behaviors. The presence of dietary-related modifiable risk factors was operationalized by using 6 indicators: students who did not eat breakfast, did not drink milk, did not eat vegetables, did not eat fruit or drink 100% fruit juice, did not drink sports drinks, and did not drink a can, bottle, or glass of soda or pop during the 7 days before the survey. The presence of physical activity–related risk factors was identified by using 5 indicators: students who did not attend physical education classes during the past week, did not participate in at least 60 minutes of physical activity on at least 1 day during the 7 days before the survey, did not play on at least 1 sports team during the past school year, played video or computer games or used computers 3 or more hours per day on an average school day, and watched television for 3 hours or more per day on an average school day. In addition, 3 other lifestyle-related risk factors were included as independent variables: students who currently drank alcohol (defined as at least 1 drink of alcohol on at least 1 day during the 30 days before the survey), currently used cigarettes, cigars, or smokeless tobacco (defined as use on at least 1 day during the 30 days before the survey), and slept 8 hours or less on an average school night (22).

Individual respondent’s demographic factors — age (in years), sex (male or female), and race/ethnicity (non-Hispanic black, non-Hispanic white, Hispanic, and other) — were used as covariates.

### Analysis

Data management and statistical analyses were performed by using SAS version 9.4. We conducted bivariate analyses by using χ^2^ tests to assess demographic and health behavior characteristics among students in grades 9 through 12. PAF and variance estimation were conducted in a 4-step procedure by using a SAS macro developed by Heeringa et al for estimating PAFs by using complex survey design data (17). In the first of the 4 steps, the risk model was identified and its parameters were estimated by using multivariable logistic regression. As part of this multivariable model, all hypothesized modifiable and nonmodifiable risk factors were entered into the model in the same step to calculate adjusted parameter estimates and odds ratios (ORs). In the second step, population-weighted PAFs were constructed. In estimating PAFs for individual modifiable risk factors, mutually exclusive scenarios were created by assuming a path in which each risk factor is the first and only one to be eliminated (23). However, students with more than 1 risk factor could prevent obesity in more than one way. Therefore, PAFs for individual risk factors often overlap and add up to more than the overall PAF estimate for all risk factors combined (23,24). In the third step, the jackknife repeated replication method was used to estimate the sampling variability of PAF point estimates, taking into account the properties of the sample design. The fourth step was to calculate confidence intervals (CIs) for PAFs. This method shows an unbiased sampling error from a complex sample survey and can account for all hypothesized confounders (17). We accounted for individual respondents’ demographic variables in all statistical models.

The YRBS complex survey design features and sampling weights were applied in all analyses to account for its complex sample design and nonresponse from schools and students. The results were also weighted to represent the total high school student population in the United States.

## Results

The final sample for analysis was a weighted sample of 15,624 respondents: 7,955 (51.3%) boys and 7,551 (48.7%) girls. Most of our study sample was non-Hispanic white (54.5%), followed by Hispanic (22.3%) and non-Hispanic black (13.6%) (Table 1). Among all respondents, 2,005 (13.9%) had obesity. A greater proportion of boys (16.8%) than girls (10.8%) had obesity. A significant proportion of high school students engaged in unhealthy dietary, physical activity, or other lifestyle behaviors. About 24.7% of students watched television for 3 hours or more per day on an average school day; 42.4% did not play on any sports team during the past school year; 18.5% currently used cigarettes, cigar, or smokeless tobacco; and 72.7% slept 8 hours or less on an average school night.

**Table 1:** Demographic Characteristics and Modifiable Risk Factors of Obesity Among High School Students, United States (N = 15,624), 2015 Youth Risk Behavior Survey

Characteristic or Modifiable Risk Factor^a^	Total, No. (%) (N = 15,624)^b^	Obese, No. (%) (n = 2,005 [13.9])	Not Obese, No. (%) (n = 12,415 [86.1])	*P* Value^c^
**Age, y**				
≤15	5,666 (36.4)	697 (15.4)	4,513 (84.6)	.28
>15	9,895 (63.6)	1,308 (14.2)	7,902 (85.8)	
**Sex**				
Male	7,955 (51.3)	1,248 (16.8)	6,168 (83.2)	<.001
Female	7,551 (48.7)	757 (10.8)	6,247 (89.2)	
**Race/ethnicity**				
Non-Hispanic white	8,336 (54.5)	982 (12.4)	6,940 (87.6)	<.001
Non-Hispanic black	2,078 (13.6)	311 (16.8)	1,542 (83.2)	
Hispanic	3,142 (22.3)	509 (16.4)	2,589 (83.6)	
Other^d^	1,482 (9.7)	173 (12.8)	1,176 (87.2)	
**Did not eat fruit or drink 100% fruit juices^e^**				
Yes	793 (5.2)	132 (19.0)	564 (81.0)	.02
No	14,541 (94.8)	1,836 (13.6)	11,656 (86.4)	
**Did not eat vegetables^e^**				
Yes	1,022 (6.7)	131 (14.7)	762 (85.3)	.58
No	14,210 (93.3)	1,826 (13.8)	11,401 (86.2)	
**Did not drink a can, bottle, or glass of soda or pop^e^**				
Yes	4,011 (26.2)	442 (11.8)	3,289 (88.2)	.01
No	11,306 (73.8)	1,525 (14.6)	8,927 (85.4)	
**Did not eat breakfast^e^**				
Yes	2,081 (13.8)	293 (15.9)	1,548 (84.1)	.04
No	12,951 (86.2)	1,634 (13.5)	10,434 (86.5)	
**Did not drink milk^e^**				
Yes	3,140 (21.5)	350 (12.3)	2,505 (87.7)	.04
No	11,433 (78.5)	1,530 (14.4)	9,114 (85.6)	
**Drank a can, bottle, or glass of a sports drink^e^**				
Yes	7,390 (57.6)	969 (14.1)	5,894 (85.9)	.10
No	5,437 (42.4)	644 (12.9)	4,343 (87.1)	
**Did not participate in at least 60 minutes of physical activity on at least 1 day^e^**				
Yes	2,182 (14.3)	304 (15.8)	1,621 (84.2)	.07
No	13,086 (85.7)	1,661 (13.6)	10,550 (86.4)	
**Did not attend physical education classes on ≥1 days during the past week**				
Yes	7,332 (48.4)	940 (13.9)	5,845 (86.1)	.89
No	7,828 (51.6)	1,011 (14.0)	6,224 (86.0)	
**Did not play on at least 1 sports team during the past school year**				
Yes	6,111 (42.4)	937 (17.1)	4,545 (82.9)	<.001
No	8,311 (57.6)	925 (11.8)	6,929 (88.2)	
**Watched television ≥3 hours per day on an average school day**				
Yes	3,720 (24.7)	592 (17.5)	2791 (82.5)	<.001
No	11,309 (75.3)	1,345 (12.8)	9,164 (87.2)	
**Played video or computer games or used a computer ≥3 hours per day on an average school day**				
Yes	6,317 (41.7)	905 (15.6)	4,909 (84.4)	<.001
No	8,826 (58.3)	1,039 (12.7)	7,151 (87.3)	
**Currently drink alcohol^f^**				
Yes	4,646 (32.8)	592 (13.6)	3,765 (86.4)	.90
No	9,553 (67.2)	1,207 (13.7)	7,606 (86.3)	
**Currently use cigarettes, cigars, or smokeless tobacco^g^**				
Yes	2,762 (18.5)	448 (17.8)	2,062 (82.2)	<.001
No	12,129 (81.5)	1,469 (13.1)	9,775 (86.9)	
**Had ≤8 hours sleep on an average school night**				
Yes	10,824 (72.7)	1,398 (13.9)	8,661 (86.1)	.80
No	4,065 (27.3)	529 (14.2)	3,208 (85.8)	

a Percentages are based on weighted data to represent all students in grades 9 through 12 attending public and private schools in the United States. Source: Centers for Disease Control and Prevention (18).

b Sum of categories in each variable does not always add up to a total sample size of 15,624 because of missing values.

c *P* values were calculated by using χ^2^ tests.

d Other race included American Indian or Alaska Native, Asian, Native Hawaiian or Other Pacific Islander, and multiple races.

e During the 7 days before the survey.

f Defined as at least 1 drink of alcohol on at least 1 day during the 30 days before the survey.

g Defined as use on at least 1 day during the 30 days before the survey.

Results of the multivariable logistic regression model show the associations between obesity and each dietary, physical activity, and other lifestyle-related risk behavior in the study sample after confounding all other variables (Table 2). After adjusting for respondents’ demographic characteristics, we found that not playing on a sports team (OR, 1.61; 95% CI, 1.31–1.98), currently using tobacco products (OR, 1.42; 95% CI, 1.14–1.77), and watching television for 3 hours or more per day (OR, 1.38; 95% CI, 1.09–1.76) were all significantly positively associated with having obesity.

**Table 2:** Multivariable Logistic Regression Determining the Odds of Having Obesity Among High School Students, United States (N = 15,624), 2015 Youth Risk Behavior Survey^a^

Modifiable Risk Factors^b^	Odds Ratio (95% Confidence Interval)
**Did not eat fruit or drink 100% fruit juices^c^**	
Yes	1.45 (0.97–2.15)
No	1 [Reference]
**Did not eat vegetables^c^**	
Yes	0.83 (0.58–1.19)
No	1 [Reference]
**Drank a can, bottle, or glass of soda or pop^c^**	
Yes	1.04 (0.8–1.34)
No	1 [Reference]
**Did not eat breakfast^c^**	
Yes	1.23 (0.97–1.57)
No	1 [Reference]
**Did not drink milk^c^**	
Yes	0.80 (0.62–1.02)
No	1 [Reference]
**Drank a can, bottle, or glass of a sports drink^c^**	
Yes	0.99 (0.86–1.16)
No	1 [Reference]
**Did not participate in at least 60 minutes of physical activity on at least 1 day^c^**	
Yes	1.06 (0.84–1.32)
No	1 [Reference]
**Did not attended physical education classes on ≥1 days during the past week**	
Yes	1.03 (0.86–1.31)
No	1 [Reference]
**Did not play on at least 1 sports team during the past school year**	
Yes	1.61 (1.31–1.98)
No	1 [Reference]
**Watched television ≥3 hours per day on an average school day**	
Yes	1.38 (1.09–1.76)
No	1 [Reference]
**Played video or computer games or used a computer ≥3 hours per day on an average school day**	
Yes	1.19 (0.98–1.43)
No	1 [Reference]
**Currently drink alcohol^d^**	
Yes	0.99 (0.83–1.18)
No	1 [Reference]
**Currently use cigarettes, cigars, or smokeless tobacco^e^**	
Yes	1.42 (1.14–1.77)
No	1 [Reference]
**Had ≤8 hours sleep on an average school night**	
Yes	1.03 (0.87–1.23)
No	1 [Reference]

a Odds ratios were estimated after accounting for the nonmodifiable risk factors age, sex, and race/ethnicity in the multivariable logistic regression model.

b Results are based on weighted data to represent all students in grades 9 through 12 attending public and private schools in the United States. Source: Centers for Disease Control and Prevention (18).

c During the 7 days before the survey.

d Defined as at least 1 drink of alcohol on at least 1 day during the 30 days before the survey.

e Defined as use on at least 1 day during the 30 days before the survey.

After controlling for respondents’ demographics, the single modifiable risk factor with the largest PAF was not playing on at least 1 sports team during the past school year, with a PAF of 16.57% (95% CI, 15.30%–17.84%) (Table 3). Other modifiable risk factors with large PAFs were watching television for 3 hours or more per day (PAF, 7.13%; 95% CI, 6.89%–7.36%), and playing video or computer games or using a computer 3 hours or more per day (PAF, 6.27%; 95% CI, 4.21%–8.32%), and currently using tobacco products (PAF, 5.73%; 95% CI, 5.07%–6.39%). In models controlling for the respondents’ age, sex, and race/ethnicity, the full PAF for all modifiable dietary, physical activity, and other lifestyle-related risk factors was 34.80% (95% CI, 32.09%–37.51%) (Table 3).

**Table 3:** Population Attributable Fractions and 95% Confidence Intervals for Modifiable Risk Factors of Obesity Among High School Students, United States (N = 15,624), 2015 Youth Risk Behavior Survey

Modifiable Risk Factors^a^	Population Attributable Fraction, % (95% Confidence Interval)
**Dietary-related risk factors**	
Did not eat fruit or drink 100% fruit juices^b^	1.53 (0.73 to 2.32)
Did not eat vegetables^b^	−1.00 (−1.26 to −0.74)
Drank a can, bottle, or glass of soda or pop^b^	2.43 (1.96 to 2.89)
Did not eat breakfast^b^	2.38 (1.80 to 2.97)
Did not drink milk^b^	−3.73 (−3.83 to −3.63)
Drank a can, bottle, or glass of a sports drink^b^	−0.05 (−0.74 to 0.64)
Dietary-related risk factors combined	2.29 (0.87 to 3.71)
**Physical activity–related risk factors**	
Did not participate in at least 60 minutes of physical activity on at least 1 day^b^	0.63 (0.32 to 0.94)
Did not attended physical education classes on ≥1 days during the past week	1.01 (−0.25 to 2.28)
Did not play on at least 1 sports team during the past school year	16.57 (15.30 to 17.84)
Watched television ≥3 hours per day on an average school day	7.13 (6.89 to 7.36)
Played video or computer games or used a computer ≥3 hours per day on an average school day	6.27 (4.21 to 8.32)
Physical activity–related risk factors combined	27.96 (26.14 to 29.78)
**Other lifestyle risk factors**	
Currently drink alcohol^c^	−0.34 (−1.15 to 0.47)
Currently use cigarettes, cigars, or smokeless tobacco^d^	5.73 (5.07 to 6.39)
Had ≤8 hours of sleep on an average school night	1.95 (1.19 to 2.70)
Other lifestyle risk factors combined	7.28 (6.51 to 8.05)
**All modifiable risk factors combined**	34.80 (32.09 to 37.51)

a Values are expressed as percentages. Percentages are based on weighted data to represent of all students in grades 9 through 12 attending public and private schools in the United States. Source: Centers for Disease Control and Prevention (18).

b During the 7 days before the survey.

c Defined as at least 1 drink of alcohol on at least 1 day during the 30 days before the survey.

d Defined as use on at least 1 day during the 30 days before the survey.

## Discussion

In the United States, data on childhood obesity and potential correlates are primarily collected in 2 cross-sectional surveys, the National Health and Nutrition Examination Survey (NHANES) and YRBS, both of which employ complex probability sample designs (1,4,16). Although statistical methodologies for computing point estimates and variances of PAFs by using cross-sectional surveys with complex sample designs have continued to develop in recent decades (13,17), major statistical software packages such as SAS and Stata still do not have the capacity to estimate PAF and variance from complex sample survey data. Our study used a methodology developed to provide useful insights into the association between modifiable risk factors and obesity among students in grades 9 through 12 in the United States (17). To our knowledge, ours is the first study to quantify the association of modifiable risk factors and childhood and adolescent obesity in the United States by using a nationally representative sample of high school students. PAFs provide a useful way to quantify the burden of obesity associated with various modifiable or nonmodifiable risk factors; however, their use in research has been limited.

Our findings were consistent with previous research showing that several unhealthy behaviors, including not playing on a sports team, watching television for 3 hours or more per day, and using tobacco were associated with obesity in high school students (20,21). Many risk factors of obesity, such as genetically determined traits (eg, age, sex, race/ethnicity) and parental factors, are not easily modifiable, so we focused on the more readily modifiable risk factors. We found that the combination of all modifiable unhealthy dietary, physical activity, and other lifestyle risk factors is associated with 34.8% of obesity in this population. This finding is striking because it shows that if all students became physically active, ate healthy foods, and adopted healthy lifestyles (such as not using tobacco and sleeping ≥8 hours per day), the prevalence of obesity in this population could be substantially reduced. We found that 42.4% of students did not participate in at least 1 sports team during the past school year; this modifiable risk factor had the most substantial contribution to obesity, with a PAF of 16.57%. Interventions coordinated at the student’s home or at high schools may start with involvement in sports teams before other risk factors are addressed.

In our study, physical activity–related risk factors combined were associated with 27.96% of the prevalence of obesity in this population, indicating that being physically active and limiting sedentary behavior in general is important in preventing obesity among high school students. A meta-analysis found that youths who participate in sports are more likely to be physically active than nonparticipants (25), but further research is needed to provide clear evidence as to what types of sports are beneficial in preventing childhood and adolescent obesity. Because of the wide variety of sports, the prevalence of obesity also varies among sports (25). The relationship between participation in various types of sports teams and the development of obesity should be carefully examined before an intervention is implemented to engage youths in sports teams.

Too much screen time, including television viewing, computer use, and videogame playing, is considered sedentary activity; television viewing, in particular, is associated with obesity among children and adolescents (21). We found that watching television or using a computer or videogame for 3 hours or more per day is linked with obesity in high school students, with a PAF of 7.1%. Children and adolescents with too much screen time may consume less energy, have less time for physical activity, and eat excessively while viewing, all of which lead to energy surplus and obesity. The results of this study help establish the priority of physical activity interventions to prevent obesity among high school students.

The benefits of physical activity in reducing obesity can be realized only when students also adopt healthy dietary habits so that they do not overcompensate for increased physical activity by eating more junk food or consuming more sugary drinks (25). Therefore, the combination of balancing caloric intake with physical activity and limiting sedentary behaviors is essential to maintaining normal growth and preventing obesity in children and adolescents (26). Although none of the dietary factors were significant in the multivariable risk estimation model used in this study, we found that dietary factors were associated with 2.3% of obesity in this population.

We observed negative PAFs for a few risk factors. According to the literature, a negative PAF indicates that the factor is protective or preventive (24,27,28). In our study, not drinking milk during the 7 days before the survey showed a negative association with having obesity, with a PAF of −3.73% (95% CI, −3.83% to −3.63%). A possible explanation is that the consumption of milk, especially whole milk or milk that contains high levels of saturated fat, may lead to childhood and adolescent obesity, because such milks contain more calories than reduced-fat or fat-free milks (26). This hypothesis is corroborated by the American Academy of Pediatrics’ recommendation that children aged 2 years or older consume skim or 1% milk rather than 2% or whole milk (26). In addition, not eating vegetables also showed a negative PAF (−1.00%; 95% CI, −1.26% to −0.74%), indicating that vegetable intake may not have weight control benefits in this population. In YRBS, the definition of vegetable includes “green salad, potatoes (excluding French fries, fried potatoes, or potato chips), carrots, and other vegetables” (22). YRBS data do not indicate the types and quantities of vegetables eaten by survey respondents. The negative association found in our study may have been caused by respondents’ consuming vegetables with a high glycemic index, such as potatoes, which are associated with weight gain (29). Nevertheless, this finding should be interpreted with caution and further research is needed on the types of vegetables consumed and their effect on childhood obesity.

Two other risk factors, the consumption of sports drinks and alcohol, also showed negative PAFs, but the confidence intervals included 0, indicating that the evidence is insufficient to determine the association between these factors and obesity in our population (13,17,30).

Among the other lifestyle-related factors evaluated in this study, we found that both tobacco use (PAF, 5.73%) and lack of sleep (PAF, 1.95%) were associated with the prevalence of obesity. However, no single modifiable behavior risk alone can explain the obesity status of children and adolescents. Comprehensive interventions that promote healthy diet, physical activity, reduced screen time, adequate sleep, and not drinking alcohol or smoking should therefore be implemented to reduce childhood obesity. The importance of a healthy lifestyle for the overall health of children and adolescents cannot be overstated. School, family, and community should share the responsibility to help promote healthy lifestyles and prevent obesity in children and adolescents.

Our study had several limitations. First, YRBS is a cross-sectional survey, and respondent’s behavioral risk factors and height and weight information were collected in 1 survey questionnaire. Therefore, the PAFs of modifiable risk factors of obesity are indicators of association, not cause and effect. Second, we did not consider all potential risk factors of obesity; we focused only on readily modifiable risk factors at the respondent level. Other risk factors, such as parental and environmental factors, may also be modifiable but were not examined in our study because such information was not available in YRBS data. Additionally, respondents’ height, weight, health behavior, and lifestyle factors were self-reported through questionnaires and thus are subject to such biases as social desirability bias and recall bias. The data were not validated by medical records, food diaries, or school records.

Physical activity, dietary, and other lifestyle factors (alcohol, tobacco, and sleep habits) were associated with over one-third of the obesity among high school students in the United States. Our study provides evidence that a substantial proportion of obesity in this population could be prevented through changes in unhealthy diet, sedentary lifestyle, and other harmful lifestyle behaviors. School, family, and community interventions focusing on promoting physical activity, healthy eating, and other healthy behaviors are important for reducing obesity and many chronic diseases in children and adolescents.
